# Studies of the Thermophysical Properties of Selected Hot-Working Tool Steels in a Wide Temperature Range

**DOI:** 10.3390/ma18040852

**Published:** 2025-02-15

**Authors:** Piotr Koniorczyk, Mateusz Zieliński, Janusz Zmywaczyk

**Affiliations:** Faculty of Mechatronics, Armament and Aerospace, Military University of Technology, ul. gen. S. Kaliskiego 2, 00-908 Warsaw, Poland; mateusz.zielinski@wat.edu.pl (M.Z.); janusz.zmywaczyk@wat.edu.pl (J.Z.)

**Keywords:** WLV (1.2365) steel, 38HMJ (1.8509) steel, WCL (1.2342) steel, thermal diffusivity, thermal expansion

## Abstract

In this work, measurements of the thermal diffusivity and thermal expansion of hot-working tool steel WLV (1.2365) were carried out in the temperature range from (−)50 °C to 500 °C. The results of these measurements were compared with those published earlier by the authors for other selected hot-working tool steels, i.e., 38HMJ (1.8509) and WCL (1.2342), in the temperature range from (−)50 °C to 500 °C and from room temperature (*RT*) to 1100 °C. This paper describes the procedures used to relate the thermal characteristics of the thermal diffusivity and thermal expansion obtained at low temperatures to those obtained at high temperatures. Thermal diffusivity and thermal expansion of WLV, 38HMJ and WCL steels in the temperature range from (−)50 °C to 1100 °C were used as input data for numerical simulations of heat transfer in devices made of these steels and operating in a wide temperature range. Thermophysical properties were tested using specialized NETZSCH test stands. Thermal diffusivity was studied using a LFA 467 light flash apparatus in the temperature range from (−)50 °C to 500 °C and a LFA 427 laser flash apparatus in the temperature range from *RT* to 1100 °C. Thermal expansion was tested using a DIL 402 Expedis dilatometer in the range from (−)50 °C to 500 °C and a DIL 402 C dilatometer in the temperature range from *RT* to 1100 °C. Finally, the results of tests on thermophysical properties of selected steels in the temperature range from (−)50 °C to 1100 °C were summarized.

## 1. Introduction

Numerical simulations are currently used in every field of technology, enabling the design of efficient and safe devices and structures. The knowledge of thermophysical properties, and in particular the thermal characteristics of the thermal diffusivity and thermal expansion of construction materials, plays a key role in obtaining accurate results of numerical simulations of heat transfer in a wide temperature range. This applies in particular to various types of steel, which are among the most common building materials in the world [1,2]. Steels are used where an appropriate strength and safety of a structure are required. An example of numerical analysis for safety reasons is the study of the influence of reinforced concrete structure architecture on the distribution of temperature fields in structural elements [3,4]. This type of analysis requires testing the steel mechanically in fire conditions as well. It is necessary to understand the mechanical damage to steel in fire. Other examples of devices that operate at high temperatures are hot extrusion dies and molds [5]. These types of components operate under very high mechanical and thermal loads. They are made of hot-work tool steels. These steels are also used to build pipes loaded with intense heat pulses, in which gas flows at a high pressure (in the order of hundreds of MPa) and high temperature (in the order of thousands of Kelvin) [6]. The thermal characteristics of thermal diffusivity and thermal expansion of three steels of this type, i.e., 38HMJ, WLV and WCL, are presented in this paper [7]. Obtaining these characteristics from sub-zero to high temperatures with a single device is often difficult because the measuring equipment is optimized for either high or low temperature operation [8,9].

The laser flash method, and the Laser Flash Apparatus (LFA) and the rod dilatometer (DIL) based on it are widely recognized as effective tools for measuring the thermal diffusivity and thermal expansion of a solid specimen in a wide temperature range [10,11,12,13,14,15]. The literature lacks descriptions of methods for combining the thermal characteristics obtained from measurements with different devices in a wide temperature range. It should be emphasized that these characteristics often do not overlap in common temperature ranges. This effect is actually due to the different accuracy of measuring a given parameter and the different aging rates of devices. This applies especially to the thermal characteristics of thermal diffusivity and, in special cases, thermal expansion [7].

Thermophysical property measurements in a wide temperature range are usually performed with at least two devices, one in the low temperature range and one in the high temperature range. This applies to measurements of thermal diffusivity and thermal expansion on Netzsch devices, e.g., for thermal diffusivity on the LFA427 and LFA467, and for thermal expansion on the DIL 402 Expedis and DIL 402C [16,17,18,19,20]. Despite the fact that the manufacturer declares the same measurement accuracy, and despite using the same measurement methods and even the same heat transfer models, there is a problem of stitching the results obtained at low temperatures with those obtained at high temperatures [17,21]. This is particularly evident in the case of thermal diffusivity, but problems also arise in the case of thermal expansion when we heat the sample several times without removing it from the dilatometer.

### 1.1. Thermal Diffusivity

**Figure 1:**
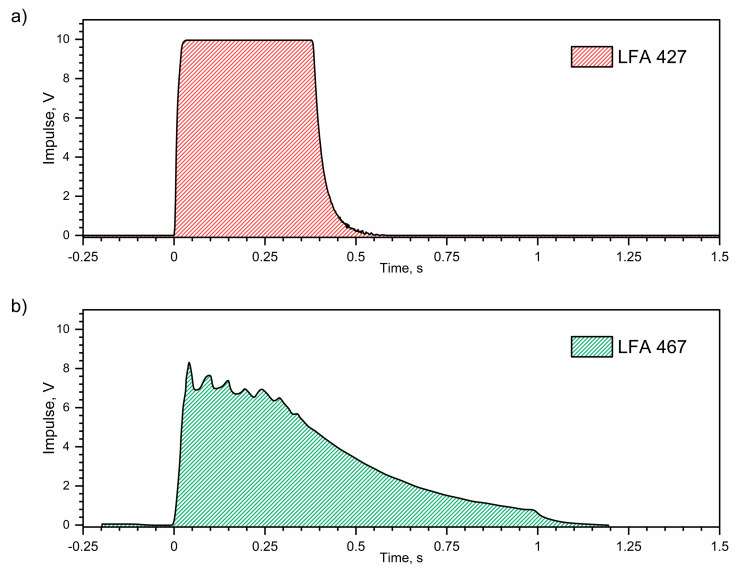
(**a**) Example of a laser pulse from the LFA 427, (**b**) example of a xenon lamp pulse from the LFA 467.

Despite the use of the same heat transfer model in the measuring specimen and the same method for determining the thermal diffusivity of the tested steels in both devices, i.e., LFA427 and LFA467, the obtained thermal characteristics of thermal diffusivity differ slightly, i.e., they do not overlap in the range from *RT* to approx. 500 °C. This is probably related to the impossible assumption of the heat transfer model in the specimen, which assumes that the front surface of the specimen is heated by a Dirac pulse at time *t* = 0 [17,19,20]. In the case of a high-temperature device, the adopted model of heat transfer in the specimen is closer to reality, because the laser pulse is, for example, 0.6 ms wide, i.e., it is short and has a shape close to a rectangle (Figure 1a). In the case of a low-temperature device, the pulse is obtained from a xenon lamp, but the shape of the pulse significantly differs from the Dirac pulse, and additionally the pulse is wide and extended in time, for example 1.2 ms (Figure 1b). It therefore seems that the results of the thermal diffusivity measurement of steel obtained using the LFA 427 device are more reliable, i.e., they are burdened with a smaller error compared to the results obtained using the LFA 467 device. The thermal diffusivity measurements of the tested steels were performed using both devices, i.e., LFA 427 and LFA 467 with the NETZSCH Proteus v.8.0.2 software and the *Cape–Lehman plus pulse correction heat transfer model* in the specimen, which takes into account the pulse width [22,23,24,25]. According to NETZSCH, the accuracy of the thermal diffusivity determination in both devices is the same and is +/− 3% [16]. According to the authors of this paper, in the case of a pulse extended in time, the results of thermal diffusivity calculations will be overestimated [26,27,28].

### 1.2. Thermal Expansivity

In heat transfer calculations, an important role is played by the thermal characteristics of steel density, calculated by the relationship [25]:

(1) $$\rho \left(T\right)=\rho_{0}\cdot {\left(1+\varepsilon \left(T\right)\right)}^{-3}$$

where $\rho_{0}$ stands for density of steel at *RT*; $\varepsilon \left(T\right)=∆L/L_{0}$ is the thermal expansion, i.e., the relative change in the length of the specimen compared to the length of the specimen at *RT*.

**Figure 2:**
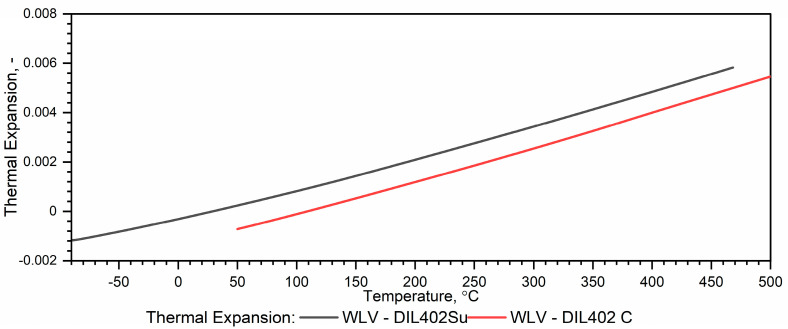
Example measurement results $\varepsilon \left(T\right)$ for the WLV hot-work tool steel obtained from DIL 402 Expedis (own results) and DIL 402 C (based on data [7,29]).

In contrast to the thermal diffusivity measurements, the thermal expansion characteristics obtained with the DIL 402 Expedis (DIL 402 Su) generally agree with the results of measurements taken with the DIL 402 C in the temperature range from *RT* to 500 °C [26,27]. Therefore, $\rho \left(T\right)$ calculations can be performed over a wide temperature range, i.e., from (−)50 °C to 1100 °C, without additional procedures related to combining both characteristics. A problem occurs when the specimen has shrunk during the first run and the $\rho \left(T\right)$ calculations are performed for the second heating run—Figure 2.

The aims of this work are as follows:

- Measure the thermal diffusivity and thermal expansion of hot-working tool steel WLV (1.2365) in the temperature range from (−)50 °C to 500 °C;
- Investigate the methods of combining the thermal diffusivity characteristics *a(T)* of WLV, 38HMJ and WCL steels obtained with both devices, i.e., low-temperature LFA 467 and high-temperature LFA 427, into thermal diffusivity characteristics in the full temperature range, i.e., from (−)50 °C to 1100 °C;
- Show the *a(T)* dependencies of selected steels as input data for calculations in the range from (−)50 °C to 1100 °C;
- Propose $\varepsilon \left(T\right)$ conversion procedures in the range from (−)50 °C to 500 °C, which shift the $\varepsilon \left(T\right)$ characteristics to point 0 for *RT;*
- Show the $\rho \left(T\right)$ dependencies of selected steels as input data for calculations in the range from (−)50 °C to 1100 °C.

The main objective of this paper is to develop methods for combining the thermal characteristics of thermal diffusivity and thermal expansion obtained from measurements with different devices in the full temperature range, i.e., from (−)50 °C to 1100 °C, because there is no description of such methods in the literature.

## 2. Materials and Methods

### 2.1. Materials

WLV steel has been the subject of research on the thermal characteristics of thermal diffusivity and thermal expansion at low temperatures, i.e., in the range from (−)50 °C to 500 °C. Previously, the authors tested the same parameters of the same steel in the range from *RT* to 1100 °C [7,29]. The same 500.0 mm long and 71.0 mm diameter WLV steel bars, manufactured by Akrostal from Rogozno, Poland, were used to make the specimens. The nominal composition (wt.%) of WLV test steel is given in Table 1. The composition was obtained using a Foundry Master Spectrophotometer 01D0058 (Optic 01D0059) [30].

As shown earlier, WLV steel is characterized by a ferrite–to–austenite transformation temperature of about 835.8 °C, which is related to the shrinkage of the steel [7].

### 2.2. Specimen Preparation

**Figure 3:**
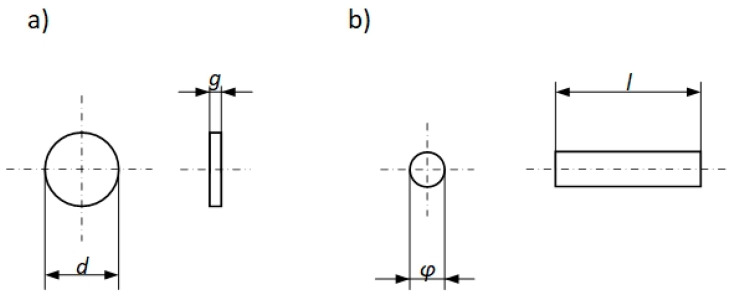
Specimens for the testing of the WLV steel: (**a**)—thermal diffusivity; (**b**)—thermal expansion.

Specimens for testing the thermophysical properties of WLV steel were prepared in the same way as previously mentioned for measurements in a high temperature range, i.e., [7,29]:

- Specimens for testing thermal diffusivity had the shape of a cylinder with a diameter of *d* = 12.70 mm and a thickness of *g* = 1.63 mm, cut from a 71.0 mm diameter bar using a water-cooled cutting disc (Figure 3a);
- Specimens for testing thermal expansion had the shape of a cylinder with a diameter of φ = 6.0 mm and a length of *l* = 24.35 mm (Figure 3b); the specimens for the DIL tests were cut from the bar using a water-cooled cutting disc.

The density of the steel at room temperature was determined by double weighing (in air and water) using the SARTORIUS MSA125P-1CE-DA analytical balance (readability [d]: 0.01 mg). The density of the WLV steel was 7.80 g/cm^3^.

### 2.3. Thermal Diffusivity and Thermal Expansion Measurements

All measurements of the thermal diffusivity and thermal expansion of WLV steel and other steels, i.e., 38HMJ and WCL, presented in this paper were performed in the laboratory of the Military University of Technology (Warsaw, Poland), which was equipped with thermal analysis devices from NETZSCH (Selb, Germany)—a world-renowned manufacturer of thermal analysis instruments.

Thermal diffusivity measurements at low temperatures, i.e., from (−)50 °C to 500 °C, were performed with a NETZSCH Light Flesh LFA 467 (Selb, Germany) using the standard Cape–Lehman model with pulse correction [25]. The front surface of the plane-parallel specimen was heated by a short energy pulse generated by a xenon lamp. From the resulting excess temperature on the rear surface measured by an IR detector, the thermal diffusivity was calculated. Argon at a flow rate of 50 $mL\cdot {min}^{-1}$ was used as an inert gas. The surface of the specimens were coated with a thin layer (2–3 μm) of graphite (GRAPHIT 33 Kontakt Chemie, Zele, Belgium) to ensure the high absorption of the pulse generated by a xenon lamp.

Thermal expansion measurements at low temperatures, i.e., from (−)50 °C to 500 °C, were performed using a NETZSCH pushrod DIL 402 Expedis dilatometer (Selb, Germany). Liquid nitrogen was used to obtain low temperatures [25]. Helium was used as an inert gas at a flow rate of 60 $mL\cdot {min}^{-1}$. Thermal expansion of the sample during heating or under isothermal conditions was detected by a displacement system to which the pusher was connected.

## 3. Results and Discussion

### 3.1. Thermal Diffusivity Results

#### 3.1.1. WLV Thermal Diffusivity Results

**Figure 4:**
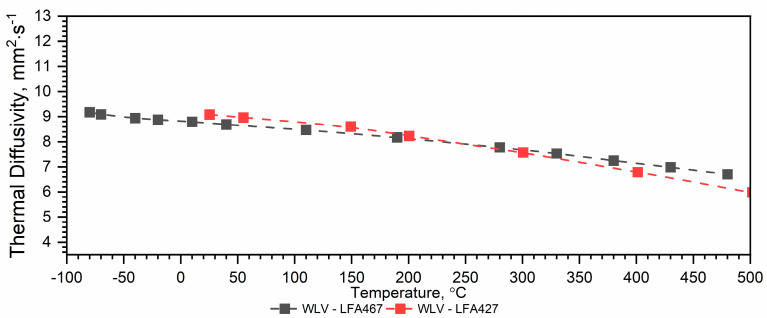
Thermal diffusivity as a function of temperature for the WLV hot-work tool steel obtained from LFA 467 (own results) and LFA 427 (based on data [7,29]).

The results of the thermal diffusivity measurements of WLV steel as a function of temperature *a(T)* in the temperature range from (−)50 °C to approximately 500 °C, performed using LFA 467, were compared with the results of such measurements carried out using LFA 427 in the same temperature range—Figure 4. The maximum relative error ∆*a*/*a* between the results from LFA 467 and LFA 427 related to the thermal diffusivity value from LFA 467 occurs at 500 °C and is 7%. Below 500 °C, i.e., in the range from *RT* to approximately 500 °C, the results from both devices are within 3%.

#### 3.1.2. Thermal Diffusivity of WLV, 38HMJ and WCL Steels in the Temperature Range from −50 °C to 1100 °C

**Figure 5:**
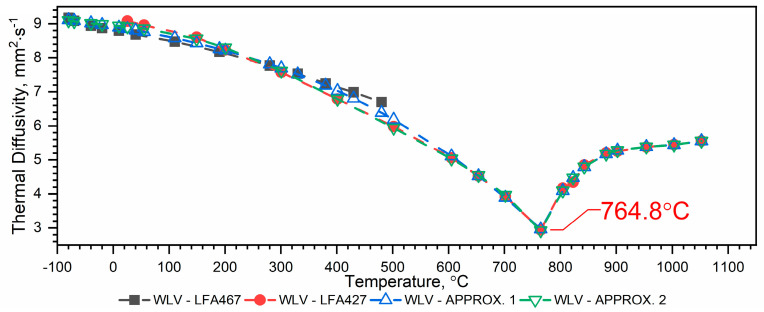
Thermal diffusivity as a function of temperature for the WLV hot-work tool steel in the range of (−)50 °C to 1100 °C ((−)50 °C–500 °C—own results, *RT*—1100 °C—based on [7,29]).

**Figure 6:**
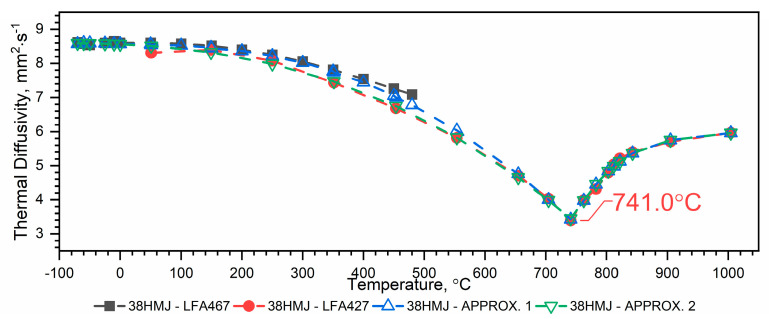
Thermal diffusivity as a function of temperature for the 38HMJ hot-work tool steel in the range of (−)50 °C to 1100 °C ((−)50 °C–500 °C—own results, *RT*—1100 °C—based on [26,27]).

**Figure 7:**
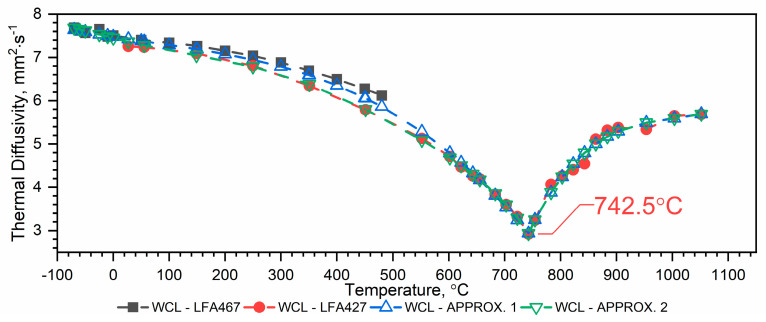
Thermal diffusivity as a function of temperature for the WCL hot-work tool steel in the range of (−)50 °C to 1100 °C ((−)50 °C–500 °C—own results, *RT*—1100 °C—based on [26,27]).

The results of the thermal diffusivity measurements *a(T)* of WLV, 38HMJ and WCL steels in the temperature range from (−)50 °C to about 1100 °C obtained using LFA 467 and LFA 427 devices are shown in Figure 5, Figure 6 and Figure 7. The combination of thermal diffusivity characteristics of WLV, 38HMJ and WCL steels obtained from both devices, i.e., low-temperature LFA 467 and high-temperature LFA 427, with thermal diffusivity characteristics in the full temperature range, i.e., from (−)50 °C to 1100 °C, can be achieved through two ways, i.e.:

1. Combine the results from LFA 467 in the range from (−)50 °C to about 500 °C with the results from LFA 427 in the range from *RT* to about 1100 °C and make an approximation in the form of a correlation formula—approx. 1 (Figure 5, Figure 6 and Figure 7, blue color);
2. The results from LFA 467 in the range from (−)50 °C to *RT* are combined with the results from LFA 427 in the range from *RT* to about 1100 °C, and the approximation is made in the form of a correlation formula—approx.2 (Figure 5, Figure 6 and Figure 7, green color).

The correlation formulas of thermal diffusivity as a function of temperature for WLV, 38HMJ and WCL steels in the form of polynomials are as follows:

For WLV steel—Figure 5:

Approx. 1—combining the data of both runs and making an approximation based on it (blue color):

(2) $$a\left(T\right)=\left\{\begin{array}{c} a_{0}+a_{1}T+a_{2}T^{2}+a_{3}T^{3},-70\;{}^{\circ}C\leq T\leq 765\;{}^{\circ}C\\ b_{0}+b_{1}T+b_{2}T^{2}+b_{3}T^{3}+b_{4}T^{4}+b_{5}\sqrt{T},\;765\;{}^{\circ}C\leq T\leq 1052\;{}^{\circ}C \end{array}\right.$$

The values of coefficients *a_i_* are given in Table 2.

Approx. 2—combining the sub-zero temperature range results from the LFA 467 with all measurement points from the LFA 427 (green color):

(3) $$a\left(T\right)=\left\{\begin{array}{c} a_{0}+a_{1}T+a_{2}T^{2}+a_{3}T^{3}+a_{4}T^{4}+a_{5}T^{5},-70\;{}^{\circ}C\leq T\leq 765\;{}^{\circ}C\\ b_{0}+b_{1}T+b_{2}T^{2}+b_{3}T^{3}+b_{4}T^{4}+b_{5}\sqrt{T},\;765\;{}^{\circ}C\leq T\leq 1052\;{}^{\circ}C \end{array}\right.$$

The values of coefficients *a_i_* are given in Table 3.

For 38HMJ steel—Figure 6:

Approx. 1—combining the data of both runs and making an approximation based on it (blue color):

(4) $$a\left(T\right)=\left\{\begin{array}{c} a_{0}+a_{1}\cdot T+a_{2}\cdot T^{2}+a_{3}\cdot T^{3}+a_{4}\cdot T^{4},-70\;{}^{\circ}C\leq T\leq 741\;{}^{\circ}C\\ b_{0}+b_{1}\cdot T+b_{2}\cdot T^{2}+b_{3}\cdot T^{3},\;741\;{}^{\circ}C\leq T\leq 1004\;{}^{\circ}C \end{array}\right.$$

The values of coefficients *a_i_* are given in Table 4.

Approx. 2—combining the sub-zero temperature range results from the LFA 467 with all measurement points from the LFA 427 (green color):

(5) $$a\left(T\right)=\left\{\begin{array}{c} a_{0}+a_{1}T+a_{2}T^{2}+a_{3}T^{3}+a_{4}T^{4}\;,-70\;{}^{\circ}C\leq T\leq 741\;{}^{\circ}C\\ b_{0}+b_{1}\cdot T+b_{2}\cdot T^{2}+b_{3}\cdot T^{3},\;741\;{}^{\circ}C\leq T\leq 1004\;{}^{\circ}C \end{array}\right.$$

The values of coefficients *a_i_* are given in Table 5.

For WCL steel—Figure 7:

Approx. 1—combining the data of both runs and making an approximation based on it (blue color):

(6) $$a\left(T\right)=\left\{\begin{array}{c} a_{0}+a_{1}T+a_{2}T^{2}+a_{3}T^{3},-70\;{}^{\circ}C\leq T\leq 743\;{}^{\circ}C\\ b_{0}+b_{1}T+b_{2}T^{2}+b_{3}T^{3}+b_{4}T^{4},\;743\;{}^{\circ}C\leq T\leq 1052\;{}^{\circ}C \end{array}\right.$$

The values of coefficients *a_i_* are given in Table 6.

Approx. 2—combining the sub-zero temperature range results from the LFA 467 with all measurement points from the LFA 427 (green color):

(7) $$\begin{array}{c} a\left(T\right)\;\\ =\left\{\begin{array}{c} a_{0}\;+\;a_{1}\;T\;+\;a_{2}\;T^{2}\;+\;a_{3}\;T^{3}\;+\;a_{4}\;T^{4}\;+\;a_{5}\;T^{5}\;+\;a_{6}\;T^{6},-70\;{}^{\circ}C\leq T\leq 743\;{}^{\circ}C\\ b_{0}+b_{1}T+b_{2}T^{2}+b_{3}T^{3}+b_{4}T^{4},\;743\;{}^{\circ}C\leq T\leq 1052\;{}^{\circ}C \end{array}\right. \end{array}$$

The values of coefficients *a_i_* are given in Table 7.

#### 3.1.3. Discussion

Both methods of determining the thermal characteristics of the thermal diffusivity of WLV, 38HMJ and WCL steels in the temperature range from (−)50 °C to 1100 °C can be used because:

1. Approx. 1—takes into account all measurement data, determined in the full temperature ranges of the thermal diffusivity measurements of both devices, i.e., from (−)50 °C to about 500 °C and from *RT* to about 1100 °C;
2. Approx. 2—takes into account measurement data determined in the temperature range from (−)50 °C to *RT* and from *RT* to about 1100 °C.

Although both methods of combining the thermal diffusivity characteristics of WLV, 38HMJ and WCL steels can be used, the authors recommend Approx. 1. The dependence of *a(T)* obtained in this way is within the measurement error for both LFA 467 and LFA 427, i.e., +/− 3%. Table 8 summarizes the relative errors of thermal diffusivity calculations $\frac{\Delta a_{app1}}{\bar{a}}$ and $\frac{\Delta a_{app2}}{\bar{a}}$ in the temperature range from (−)50 °C to approximately 500 °C, using both approximation methods, i.e., Approx. 1 and Approx. 2.

The thermal diffusivity characteristics of the tested steels, i.e., WLV, 38HMJ and WCL—Figure 5, Figure 6 and Figure 7, show that at temperatures of approx. 764.8 °C for WLV steel, 741.0 °C for 38HMJ steel and 742.5 °C for WCL steel, a phase transition from the ferromagnetic to the paramagnetic state occurs—the so-called Curie point. This was discussed in the authors’ papers [7,26,27,29]. This work describes the procedures used to relate the thermal diffusivity characteristics obtained at low temperatures to those obtained at high temperatures.

### 3.2. Thermal Expansion Results

#### 3.2.1. WLV Thermal Expansion Results

Using the NETZSCH Proteus v.8.0.2 software supporting the DIL 402 C dilatometer, we can obtain delta specimen length increments, i.e., $∆L\left(T_{i}\right)$, over the entire measured temperature range. Therefore, at each point $T_{i}$ we can write the expression for ${CLTE}^{*}(T_{i})$ as follows [25]:

(8) $${CLTE}^{*}(T_{i})=\frac{1}{L_{0}}\frac{L\left(T_{i+1}\right)-L\left(T_{i}\right)}{T_{i+1}-T_{i}}$$

because in addition we have

(9) $$∆L\left(T_{i+1}\right)=L\left(T_{i+1}\right)-L_{0}$$

(10) $$∆L\left(T_{i}\right)=L\left(T_{i}\right)-L_{0}$$

from here we obtain the expression for

(11) $$∆L\left(T_{i+1}\right)={CLTE}^{*}\left(T_{i}\right)\cdot L_{0}\cdot \left(T_{i+1}-T_{i}\right)+∆L\left(T_{i}\right)$$

**Figure 8:**
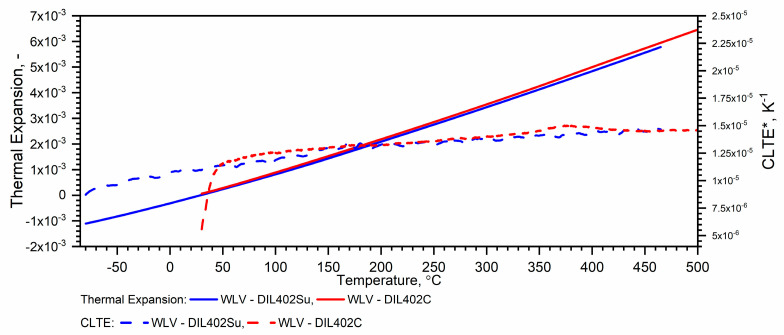
Example measurement results $\varepsilon \left(T\right)$ and CLTE* with DIL 402 Expedis and DIL 402 C ((−)50 °C–500 °C—own results, RT—1100 °C—based on [7,29]).

If in the thermal expansion characteristics in the temperature range from RT to 1100 °C $\varepsilon \left(T\right)=∆L/L_{0}$ starts below zero—Figure 2, we can recalculate its values using the expression (11)*—*Figure 8. The $\varepsilon \left(T\right)=∆L/L_{0}$ data prepared in this way can be used to calculate $\rho \left(T\right)=\rho_{0}\cdot {\left(1+\varepsilon \left(T\right)\right)}^{-3}$ over the entire temperature range, i.e., from (−)50 °C to 1100 °C—Figure 12.

#### 3.2.2. Thermal Expansion of WLV, 38HMJ and WCL Steels in the Temperature Range from −50 °C to 1100 °C

**Figure 9:**
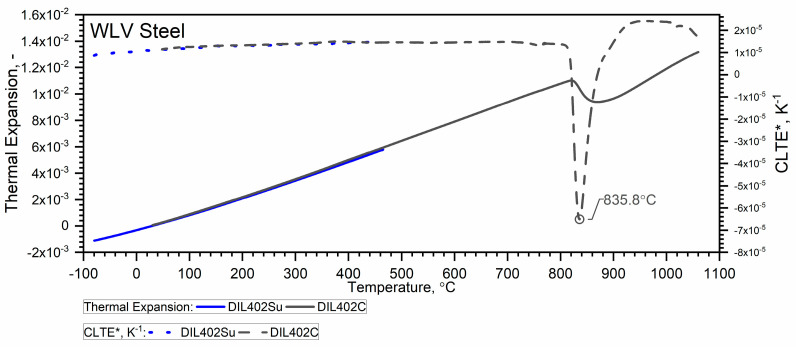
Thermal expansion $\varepsilon \left(T\right)$ and linear thermal expansion CLTE*(T) for the WLV steel ((−)50 °C–500 °C—own results, *RT*—1100 °C—based on [7,29]).

**Figure 10:**
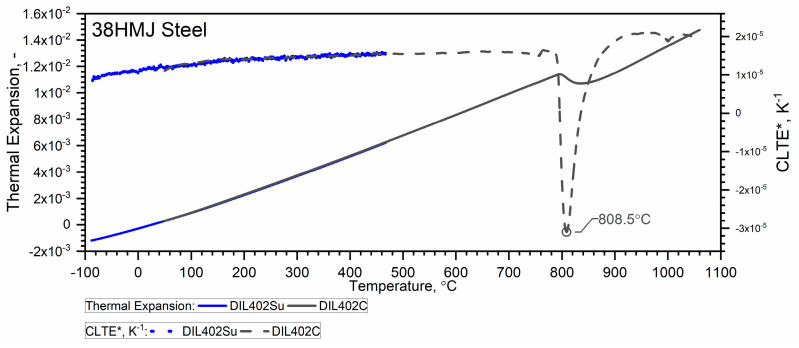
Thermal expansion $\varepsilon \left(T\right)$ and CLTE*(T) for the 38HMJ steel ((−)50 °C–500 °C—own results, *RT*—1100 °C—based on [26,27]).

**Figure 11:**
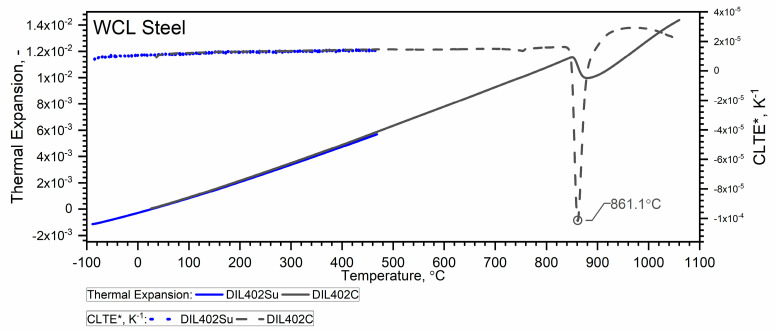
Thermal expansion $\varepsilon \left(T\right)$ and CLTE*(T) for the WCL steel ((−)50 °C–500 °C—own results, *RT*—1100 °C—based on [26,27]).

**Figure 12:**
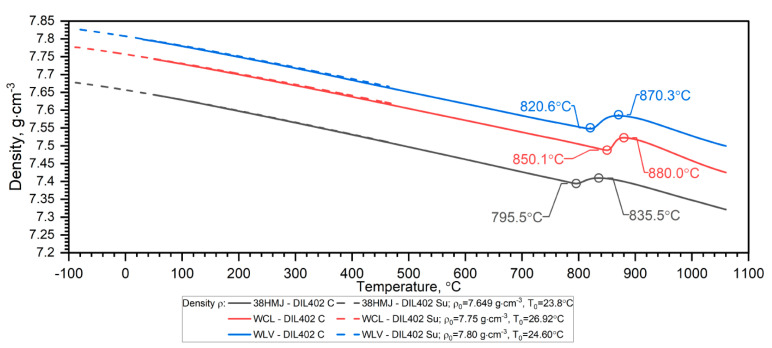
Density $\rho \left(T\right)$ for the WLV, 38HMJ and WCL steels ((−)50 °C–500 °C—own results, *RT*—1100 °C—based on [7,26,27,29]).

Due to the material shrinkage that occurs in the tested steels, and which is characterized by a rapid increase in density—Figure 12, it is more convenient to present the $\rho \left(T\right)$ characteristics in the form of data densification at the shrinkage temperature. The results of the thermal expansion measurements $\varepsilon \left(T\right)$ and the coefficient of linear thermal expansion (CLTE) regarding the initial specimen length $L\left(T_{0}\right)=L_{0}$—denoted as ${CLTE}^{*}\left(T\right)=\frac{1}{L_{0}}\frac{dL\left(T\right)}{dT}$ of WLV, 38HMJ and WCL steels in the temperature range from (−)50 °C to about 1100 °C obtained using DIL 402 Expedis (DIL 402 Su) and DIL 402C devices are shown in Figure 9, Figure 10, Figure 11 and Figure 12 and summarized in Table 9.

The thermal expansion characteristics of the tested steels, i.e., WLV, 38HMJ and WCL—Figure 9, Figure 10 and Figure 11, show that at a temperature of about 835.8 °C for WLV steel, 808.4 °C for 38HMJ steel and 760.9 °C for WCL steel, a ferrite–austenite phase transition occurs—the so-called shrinkage effect. This was discussed in the authors’ work [7,26,27,29]. This paper describes the procedures used to relate the thermal expansion characteristics obtained at low temperatures to those obtained at high temperatures.

## 4. Conclusions

The authors performed measurements of the thermal characteristics of thermal diffusivity and thermal expansion of WLV steel in the low temperature range, i.e., from (−)50 °C to 500 °C. The measurement data *a(T)* and *ε(T)* for WLV steel were supplemented with the low temperature range, because the authors had previously performed such measurements in the range from *RT* to 1100 °C. In the case of the remaining steels, i.e., 38HMJ and WCL, the authors performed such tests earlier in the full temperature range, i.e., from (−)50 °C to 1100 °C. However, the methods of combining the thermal characteristics of *a(T)* and *ε(T)* in the low temperature range with the same characteristics in the high temperature range were not analyzed. Finally, the thermal characteristics *a(T)* and *ε(T)* were presented as input data for numerical simulations of heat transfer in devices made of these steels and operating in a wide temperature range. Due to the relatively mild nature of the thermal diffusivity changes as a function of temperature, the characteristic *a(T)* of selected steels, i.e., WLV, 38HMJ and WCL, were presented in the form of polynomials. In the case of the thermal characteristics of thermal expansion, it is more convenient to present the *ε(T)* data in the form of tables due to the rapid nature of the *ε(T)* changes at the shrinkage temperature of these steels, i.e., at 835.8 °C for WLV steel, 808.4 °C for 38HMJ steel and 860.9 °C for WCL steel.

This paper presents two alternative methods of combining thermal characteristics *a(T)* obtained from measurements with LFA 467—in the low temperature range, with the same results *a(T)* obtained from measurements with LFA 427—in the high temperature range. Although both methods of combining the thermal diffusivity characteristics of WLV, 38HMJ and WCL steels can be used, i.e., Approx. 1 and Approx. 2, in the temperature range from (−)50 °C to 1100 °C the authors recommend Approx. 1. We consider the full range of measurement data from both devices, and furthermore the *a(T)* relationship obtained in this way is within the measurement error limits for both thermal diffusivity characteristics, i.e., those obtained using LFA 467 and LFA 427. In the case of Approx. 1, the relative errors of thermal diffusivity calculations $\frac{\Delta a_{app1}}{\bar{a}}$ in the temperature range from (−)50 °C to approx. 500 °C are smaller compared to $\frac{\Delta a_{app2}}{\bar{a}}$.

In the case of the thermal expansion measurements of our selected steels, the thermal characteristics *ε(T)* obtained from measurements with the DIL 402 Expedis—in the low temperature range, generally coincide with the same *ε(T)* results obtained from measurements with the DIL 402 C—in the high temperature range from *RT* to 500 °C. A problem occurs when we analyze the *ε(T)* measurement results in the range from *RT* to 1100 °C from the second heating run without removing the specimen from the DIL 402 C dilatometer. Due to specimen shrinkage after the first heating, the thermal *ε(T)* characteristics start with negative values. In this case, the authors suggest calculating the *ε(T)* dependence according to the procedure proposed in this paper. It should be emphasized that material shrinkage is caused by cracks. Exceeding the shrinkage temperature multiple times results in material degradation. The described effect occurs in devices made of such steel, which operate in a wide temperature range.

The results of the thermal property studies are summarized as follows:

(1) The analysis of combining thermal characteristics *a(T)* obtained from measurements with LFA 467—in the low temperature range, with the same results *a(T)* obtained from measurements with LFA 427—in the high temperature range allows us to conclude the following:
(a) The thermal diffusivity characteristics of LFA 427 and LFA 467 in the range from *RT* to about 500 °C do not overlap. This is due to the different methods of generating a thermal pulse on the front surface of the specimen;
(b) Although both methods of combining thermal diffusivity characteristics, i.e., Approx. 1 and Approx 2, can be used, the authors recommend Approx. 1 because the *a(T)* dependence obtained in this way is within the measurement errors for both LFA 467 and LFA 427.
(2) The analysis of the DIL thermograms obtained from measurements with DIL 402 Expedis (DIL 402 Su)—in the low temperature range and DIL 402C—in the high temperature range shows the following:
(a) The thermal expansion *ε(T)* obtained by DIL 402 Expedis is substantially consistent with the *ε(T)* results obtained by DIL 402 C in the range from *RT* to 500 °C;
(b) Due to the sample shrinkage after the first heating, in the process of combining the characteristics from DIL 402 Expedis and DIL 402 C, the authors propose the calculation of the dependence *ε(T)* from DIL 402 C in accordance with the proposed procedure.

## Figures and Tables

**Table 1:** Chemical composition of the hot-work tool (WLV) steel [5,7,29,30,31].

Component	Fe	C	Si	Mn	Cr	Mo	V	P
Concentration [wt.%]	92.88	0.32	0.25	0.30	3.00	2.75	0.55	0.03

**Table 2:** Coefficients for calculating thermal diffusivity of WLV specimens in Equation (2).

−70 °C≤T≤765 °C	765 °C≤T≤1052 °C
$a_{0}=8.918838$	$b_{0}=1.480743\times 10^{4}$
$a_{1}=-2.669561\times 10^{-3}$	$b_{1}=6.917149\times 10^{1}$
$a_{2}=-3.311047\times 10^{-6}$	$b_{2}=-3.951779\times 10^{-2}$
$a_{3}=-4.423724\times 10^{-9}$	$b_{3}=1.775115\times 10^{-5}$
	$b_{4}=-3.509816\times 10^{-9}$
	$b_{5}=-1.856163\times 10^{3}$

**Table 3:** Coefficients for calculating thermal diffusivity of WLV specimens in Equation (3).

−70 °C≤T≤765 °C	765 °C≤T≤1052 °C
$a_{0}=8.952024$	$b_{0}=1.480743\times 10^{4}$
$a_{1}=-1.473539\times 10^{-3}$	$b_{1}=6.917149\times 10^{1}$
$a_{2}=-1.499612\times 10^{-6}$	$b_{2}=-3.951779\times 10^{-2}$
$a_{3}=-6.088941\times 10^{-8}$	$b_{3}=1.775115\times 10^{-5}$
$a_{4}=1.363608\times 10^{-10}$	$b_{4}=-3.509816\times 10^{-9}$
$a_{5}=-8.958574\times 10^{-14}$	$b_{5}=-1.856163\times 10^{3}$

**Table 4:** Coefficients for calculating thermal diffusivity of 38HMJ specimens in Equation (4).

−70 °C≤T≤741 °C	741 °C≤T≤1004 °C
$a_{0}=8.564750\times 10^{0}$	$b_{0}=-1.888804\times 10^{2}$
$a_{1}=-4,370618\times 10^{-4}$	$b_{1}=6.085430\times 10^{-1}$
$a_{2}=1.064581\times 10^{-7}$	$b_{2}=-6.354883\times 10^{-4}$
$a_{3}=-1.928650\times 10^{-8}$	$b_{3}=2.217722\times 10^{-7}$
$a_{4}=9.802034\times 10^{-12}$	

**Table 5:** Coefficients for calculating thermal diffusivity of 38HMJ specimens in Equation (5).

−70 °C≤T≤741 °C	741 °C≤T≤1004 °C
$a_{0}=8.563224\times 10^{0}$	$b_{0}=-1.888804\times 10^{2}$
$a_{1}=-7.107813\times 10^{-4}$	$b_{1}=6.085430\times 10^{-1}$
$a_{2}=-5.335197\times 10^{-6}$	$b_{2}=-6.354883\times 10^{-4}$
$a_{3}=-4.529680\times 10^{-9}$	$b_{3}=2.217722\times 10^{-7}$
$a_{4}=6.129077\times 10^{-13}$	

**Table 6:** Coefficients for calculating thermal diffusivity of WLV specimens in Equation (6).

−70 °C≤T≤743 °C	743 °C≤T≤1052 °C
$a_{0}\;=\;7.478481$	$b_{0}=-8.852824\times 10^{1}$
$a_{1}=-2.059501\times 10^{-3}$	$b_{1}=2.333999\times 10^{-1}$
$a_{2}=2.308221\times 10^{-6}$	$b_{2}=-1.414989\times 10^{-4}$
$a_{3}=-1.047359\times 10^{-8}$	$b_{3}=-4.244723\times 10^{-8}$
	$b_{4}=4.466081\times 10^{-11}$

**Table 7:** Coefficients for calculating thermal diffusivity of 38HMJ specimens in Equation (7).

−70 °C≤T≤743 °C	743 °C≤T≤1052 °C
$a_{0}=7.447098$	$b_{0}=-8.852824\times 10^{1}$
$a_{1}=-3.308068\times 10^{-3}$	$b_{1}=2.333999\times 10^{-1}$
$a_{2}=3.129538\times 10^{-6}$	$b_{2}=-1.414989\times 10^{-4}$
$a_{3}=2.974702\times 10^{-8}$	$b_{3}=-4.244723\times 10^{-8}$
$a_{4}=-2.023312\times 10^{-10}$	$b_{4}=4.466081\times 10^{-11}$
$a_{5}=3.502045\times 10^{-13}$	
$a_{6}=-1.998939\times 10^{-16}$	

**Table 8:** The relative errors of thermal diffusivity calculations $\frac{\Delta a_{app1}}{\bar{a}}$ and $\frac{\Delta a_{app2}}{\bar{a}}$ of selected steels in the temperature range from about (−)50 °C to about 500 °C: $a_{1}$—thermal diffusivity with LFA 467; $a_{2}$—thermal diffusivity with LFA 427; $\bar{a}=\left(a_{1}+a_{2}\right)/2$; Approx. 1—app1; Approx. 2—app2; $\Delta a_{app1}=a_{app1}-\bar{a}$; $\Delta a_{app2}=a_{app2}-\bar{a}$.

WLV				38HMJ				WCL			
T [°C]	$\bar{a}$	$\frac{\Delta a_{app1}}{\bar{a}}$	$\frac{\Delta a_{app2}}{\bar{a}}$	T [°C]	$\bar{a}$	$\frac{\Delta a_{app1}}{\bar{a}}$	$\frac{\Delta a_{app2}}{\bar{a}}$	T [°C]	$\bar{a}$	$\frac{\Delta a_{app1}}{\bar{a}}$	$\frac{\Delta a_{app2}}{\bar{a}}$
−80	9.17	0.6%	0.8%	−70	8.41	2.0%	2.2%	−70	7.45	2.6%	3.1%
−70	9.14	0.6%	0.8%	−60	8.39	2.5%	2.3%	−60	7.44	2.3%	2.8%
−40	9.07	0.5%	0.6%	−50	8.38	2.5%	2.4%	−50	7.43	2.1%	2.5%
−20	9.02	0.5%	0.4%	−25	8.38	2.4%	2.3%	−25	7.41	1.6%	1.6%
10	8.94	0.5%	0.0%	−10	8.39	2.2%	2.1%	−10	7.40	1.4%	1.1%
25	8.90	0.6%	0.2%	0	8.40	2.0%	1.9%	0	7.39	1.2%	0.8%
40	8.86	0.6%	0.5%	50	8.46	1.2%	0.7%	27	7.36	0.9%	0.0%
55	8.81	0.6%	0.5%	51	8.46	1.0%	0.6%	50	7.33	0.6%	0.4%
110	8.62	0.5%	0.7%	100	8.49	0.4%	0.6%	55	7.33	0.6%	0.7%
149	8.45	0.3%	1.2%	149	8.46	0.1%	1.6%	100	7.26	0.3%	1.1%
190	8.26	0.1%	0.7%	150	8.46	0.2%	1.6%	149	7.17	0.2%	1.7%
201	8.20	0.2%	1.2%	200	8.35	0.2%	2.1%	150	7.17	0.2%	1.6%
280	7.75	0.9%	0.3%	250	8.16	0.7%	2.2%	200	7.05	0.3%	1.8%
301	7.62	1.0%	0.1%	250	8.16	0.4%	2.2%	249	6.91	0.5%	1.8%
330	7.43	1.2%	0.3%	300	7.92	1.3%	2.1%	250	6.91	0.5%	2.0%
380	7.11	1.1%	1.2%	350	7.63	1.8%	2.0%	300	6.74	0.7%	2.2%
401	6.96	0.9%	2.5%	351	7.62	1.6%	2.0%	350	6.53	0.9%	2.5%
430	6.77	0.6%	2.7%	400	7.31	1.8%	2.2%	351	6.53	0.9%	2.3%
480	6.43	0.6%	4.7%	450	6.98	1.0%	3.0%	400	6.30	0.8%	2.9%
				453	6.96	0.7%	3.0%	450	6.03	0.5%	3.5%
				480	6.78	0.0%	3.8%	451	6.03	0.5%	3.7%
								480	5.86	0.1%	4.0%

**Table 9:** Density of WLV, 38HMJ and WCL steels in the temperature range from (−)50 °C to 1100 °C.

WLV		38HMJ		WCL	
*T*, [°C]	$\rho ,[g\cdot cm^{-3}]$	*T*, [°C]	$\rho ,[g\cdot cm^{-3}]$	*T*, [°C]	$\rho ,[g\cdot cm^{-3}]$
−85	7.825	−85	7.677	−85	7.776
−80	7.824	−80	7.676	−80	7.775
−75	7.823	−75	7.675	−75	7.774
−50	7.818	−50	7.669	−50	7.769
−25	7.812	−25	7.663	−25	7.763
0	7.806	0	7.657	0	7.757
25	7.799	25	7.650	25	7.750
50	7.793	50	7.643	50	7.744
100	7.779	100	7.629	100	7.729
200	7.750	200	7.597	250	7.685
400	7.686	400	7.531	350	7.653
600	7.620	600	7.462	500	7.604
780	7.562	780	7.399	700	7.538
800	7.555	795	7.394	800	7.505
820	7.550	800	7.395	850	7.488
830	7.557	820	7.407	858	7.496
840	7.571	830	7.409	863	7.507
850	7.58	840	7.409	869	7.518
860	7.586	850	7.408	875	7.522
870	7.587	860	7.406	882	7.523
890	7.584	870	7.403	890	7.521
920	7.573	880	7.400	902	7.517
955	7.554	890	7.396	930	7.503
1000	7.531	900	7.392	980	7.471
1020	7.520	1000	7.347	1020	7.446
1060	7.502	1060	7.321	1056	7.427

## Data Availability

The original contributions presented in this study are included in the article. Further inquiries can be directed to the corresponding author.
